# Expression profiles analysis of long non-coding RNAs identified novel lncRNA biomarkers with predictive value in outcome of cutaneous melanoma

**DOI:** 10.18632/oncotarget.20780

**Published:** 2017-09-08

**Authors:** Xu Ma, Zhijuan He, Ling Li, Daping Yang, Guofeng Liu

**Affiliations:** ^1^ Department of Plastic Surgery, The Second Affiliated Hospital of Harbin Medical University, Harbin 150086, China; ^2^ Department of Obstetrics and Gynecology, The First Affiliated Hospital of Harbin Medical University, Harbin 150010, China; ^3^ Department of Cardiology, The Second Affiliated Hospital of Harbin Medical University, Harbin 150086, China

**Keywords:** cutaneous melanoma, long non-coding RNA, overall survival, prognosis

## Abstract

Recent advancements in cancer biology have identified a large number of lncRNAs that are dysregulated expression in the development and tumorigenesis of cancers, highlighting the importance of lncRNAs as a key player for human cancers. However, the prognostic value of lncRNAs still remains unclear and needs to be further investigated. In the present study, we aim to assess the prognostic value of lncRNAs in cutaneous melanoma by integrated lncRNA expression profiles from TCGA database and matched clinical information from a large cohort of patients with cutaneous melanoma. We finally identified a set of six lncRNAs that are significantly associated with survival of patients with cutaneous melanoma. A linear combination of six lncRNAs (*LINC01260, HCP5, PIGBOS1, RP11-247L20.4, CTA-292E10.6* and *CTB-113P19.5*) was constructed as a six-lncRNA signature which classified patients of training cohort into the high-risk group and low-risk group with significantly different survival time. The prognostic value of the six-lncRNA signature was validated in both the validation cohort and entire TCGA cohort. Moreover, the six-lncRNA signature is independent of known clinic-pathological factors by multivariate Cox regression analysis and demonstrated good performance for predicting three- and five-year overall survival by time-dependent receiver operating characteristic (ROC) analysis. Our study provides novel insights into the molecular heterogeneity of cutaneous melanoma and also shows potentially important implications of lncRNAs for prognosis and therapy for cutaneous melanoma.

## INTRODUCTION

Skin cancer is the most common form of cancer and can be divided into three histological subtypes: basal cell carcinoma (BCC), squamous cell carcinoma (SCC) and cutaneous melanoma (CM) [1]. Although BCC and SCC are more common kinds of skin cancer, CM accounting for less than 5% of all skin cancer is the most aggressive skin cancer and causes more than 75% of skin cancer-related deaths worldwide [2]. The patients with localized melanoma often can be cured by surgical management alone and have good prognosis. However, 10%-40% of patients diagnosed with localized lesions still die from melanoma [3]. The current prognosis evaluation is mostly based on clinical and histological features, such as tumor thickness, mitotic rate, and ulceration which has been shown to be independent predictors of survival [4]. Recent large-scale genomic analyses have demonstrated the molecular heterogeneity of cutaneous melanoma, leading to a critical need to identify molecular markers for more accurate individualized prognosis for cutaneous melanoma patients and improve overall survival outcome [5].

With the application of whole genome and transcriptome sequencing technologies, it has been shown that only < 2% of the human genome can encode protein-coding genes and at least 90% of the genome is actively transcribed and give rise to a range of the non-coding RNA transcripts (ncRNAs) [6]. NcRNAs can be grouped into two major classes according to their size: small ncRNAs (such as microRNAs) and long non-coding RNAs (lncRNAs). Currently, lncRNAs were generally defined as mRNA-like non-coding transcripts ranging in length from 200 nt to ~100 kilobases [7]. Like mRNAs, lncRNAs are transcribed by RNA polymerase II (RNA pol II) and have a 5’terminal methylguanosine cap and are often spliced and polyadenylated [8]. Recent advancements in cancer biology have identified a large number of lncRNAs that are dysregulated expression in the development and tumorigenesis of cancers, highlighting the importance of lncRNAs as a key player for human cancers [9–11]. Several research groups have focused on expression change of lncRNAs and identified some differentially expressed lncRNAs implicated in the pathogenesis of cutaneous melanoma, revealing the potential of lncRNAs as biomarkers or therapeutic targets for cutaneous melanoma [12, 13]. Although many efforts have been made to identify lncRNA signature in a wide range of human cancers [14–31], the prognostic value of lncRNAs still remains unclear and need to be further investigated.

In this study, we analyzed the lncRNA expression profiles and clinical data in 225 patients with cutaneous melanoma from The Cancer Genome Atlas (TCGA) project. The aim of our study was to assess the prognostic value of lncRNAs in cutaneous melanoma and tried to identify a lncRNA signature that could be used as a molecular prognostic marker for patients with cutaneous melanoma.

## RESULTS

### Identification of prognostic lncRNAs from the training cohort

All patients from TCGA were first randomly divided into the training cohort (n=113) and validation cohort (n=112) for the purpose of discovery and validation of prognostic RNAs. By subjecting lncRNA expression of patients in the training cohort to univariable Cox proportional hazards regression analysis, we identified 40 prognostic lncRNA candidates that were significantly correlated with survival time (p<0.005). Then we performed the multivariate analysis to evaluate the independent prognostic value of these 40 candidate prognostic lncRNAs and identified six lncRNAs as independent prognostic factors with the ability to predict the outcome. The overview of these six prognostic lncRNAs was listed in Table 1.

**Table 1:** Overview of prognostic lncRNAs identified in the training cohort

Ensembl ID	Gene name	Chromosome	Coefficient	Hazard ratio	p-value
ENSG00000132832	LINC01260	Chromosome 20: 44,656,451-44,696,096 reverse strand	-0.325	0.722	0.005
ENSG00000206337	HCP5	Chromosome 6: 31,400,702-31,477,506 forward strand	-0.219	0.803	<0.001
ENSG00000225973	PIGBOS1	Chromosome 15: 55,317,184-55,319,161 reverse strand.	-0.683	0.505	0.005
ENSG00000226471	CTA-292E10.6	Chromosome 22: 28,800,683-28,848,559 forward strand	-0.522	0.593	<0.001
ENSG00000259071	RP11-247L20.4	Chromosome 14: 50,326,526-50,327,909 reverse strand.	-0.949	0.387	<0.001
ENSG00000272112	CTB-113P19.5	Chromosome 5: 151,724,831-151,725,356 reverse strand.	-0.463	0.629	<0.001

### Development of prognostic signature based on the six prognostic lncRNAs in the training cohort

**Figure 1:**
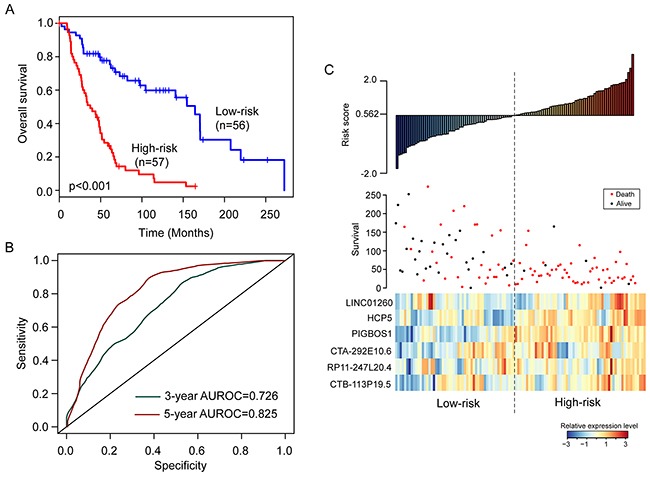
The six-lncRNA signature in the training cohort **(A)** Kaplan-Meier survival curves of overall survival between high-risk group and low-risk group. **(B)** The prediction performance for three- and five-year overall survival by the ROC analysis. **(C)** Distribution of risk scores, patient survival status and lncRNA expression map.

To construct a prognostic signature, the six prognostic lncRNAs were fitted in a multivariate Cox regression analysis in the training cohort. Then a prognostic lncRNA signature with six prognostic lncRNAs was constructed using a mathematical formula for survival prediction according to the expression of the six prognostic lncRNAs and using the multivariate Cox regression coefficient as the weight, as follows: Risk score= (-0.1779* expression value of *LINC01260*)+(-0.1522*expression of HCP5)+ (0.2537* expression value of *PIGBOS1*)+(-0.4409*expression of *CTA-292E10.6*)+ (-0.8444* expression value of *RP11-247L20.4*)+(-0.2056*expression of *CTB-113P19.5*). A patient with six-lncRNA signature risk score larger than the median risk score (0.562) was classified as high-risk, whereas patients with risk score six-lncRNA signature risk score smaller than the median risk score (0.562) was classified as low-risk. When the six-lncRNA signature was applied to the training cohort, all patients of the training cohort were divided into the high-risk group (n=57) and low-risk group (n=56) according to the threshold of the median risk score (0.562). Patients in the high-risk group had significantly shorter median survival time than those in the low-risk group (median survival 37.5 months vs. 164.3 months, p<0.001) (Figure 1A). In details, the survival of patients in the low-risk group was 81.8% at 36 months and 77.7% at 60 months which compared with 50.9% and 26.6%, respectively, in the high-risk group. The univariable analysis also revealed a significant association between six-lncRNA signature risk score and overall survival (Table 2). Time-dependent ROC curves were used to assess the prognostic power of the six-lncRNA signature. The AUC of the six-lncRNA signature for survival prediction was 0.726 at 36 months of OS and 0.825 at 60 months of OS.

**Table 2:** Univariable and multivariable Cox regression analysis of the lncRNA signature and overall survival in the training cohort (n=113), in the validation cohort (n=112) and in the combined cohort (n=225)

Variables	Univariable analysis		Multivariable analysis	
	HR (95% CI)	P value	HR (95% CI)	P value
**Training cohort (n=113)**				
LncRNA signature	2.718 (1.916-3.857)	<0.001	3.518 (2.331-5.310)	<0.001
Age	1.021 (1.004-1.039)	0.019	1.011 (0.992-1.031)	0.253
Gender(male/female)	1.214 (0.729-2.023)	0.458	1.259 (0.705-2.249)	0.437
Stage II	1.889 (0.881-4.053)	0.102	1.864 (0.861-4.039)	0.114
Stage III	2.412 (1.214-4.792)	0.012	2.214 (1.098-4.467)	0.026
Stage IV	4.677 (0.584-37.467)	0.146	21.13 (2.274-196.386)	0.007
**Testing cohort (n=112)**				
LncRNA signature	1.434 (1.091-1.885)	0.01	1.62 (1.178-2.231)	0.003
Age	1.027 (1.009-1.044)	0.002	1.02 (1.004-1.042)	0.018
Gender(male/female)	1.237 (0.745-2.056)	0.411	1.17 (0.658-2.069)	0.599
Stage II	1.3297 (0.646-2.737)	0.439	1.00(0.472-2.133)	0.993
Stage III	1.443 (0.737-2.824)	0.285	1.61 (0.805-3.234)	0.178
Stage IV	2.168 (0.775-6.063)	0.14	2.43 (0.853-6.943)	0.096
**Combined cohort (n=225)**				
LncRNA signature	1.794 (1.453-2.216)	<0.001	2.043 (1.616-2.583)	<0.001
Age	1.024 (1.012-1.036)	<0.001	1.017 (1.003-1.03)	0.014
Gender(male/female)	1.230 (0.868-1.769)	0.237	1.219 (0.817-1.819)	0.333
Stage II	1.522 (0.912-2.540)	0.108	1.188(0.693-2.038)	0.531
Stage III	1.786 (1.118-2.854)	0.015	1.926 (1.196-3.102)	0.007
Stage IV	2.672 (1.096-6.513)	0.031	3.21 (1.298-7.941)	0.012

Distribution of the six-lncRNA signature risk scores, the expression pattern of prognostic lncRNAs and the survival status was shown in Figure 1C. As shown in Figure 1C, the higher levels of expression of six lncRNAs in the signature were associated with shorter survival of patients.

### Validation of the six-lncRNA signature for survival prediction in the validation cohort and entire TCGA cohort

**Figure 2:**
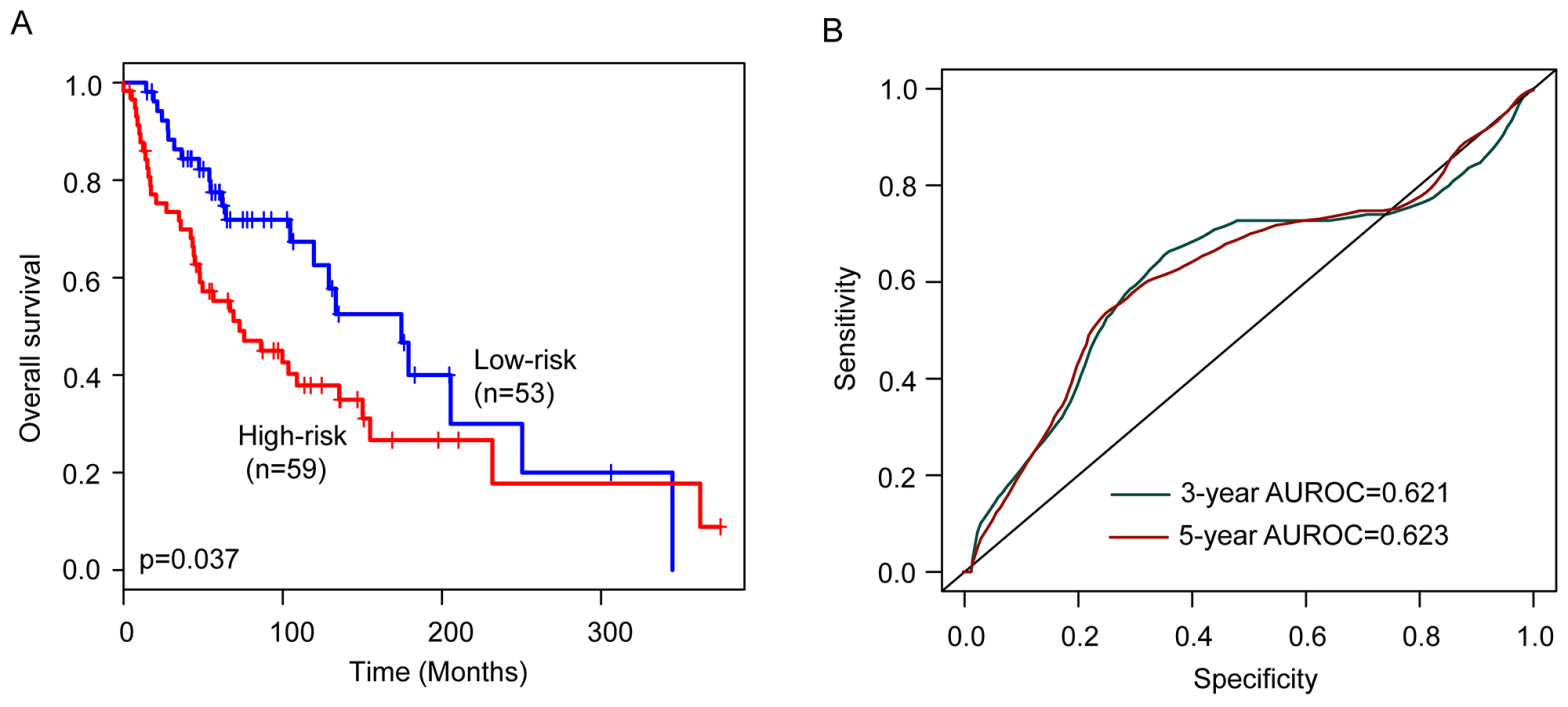
Performance validation of the six-lncRNA signature in the validation cohort **(A)** Kaplan-Meier survival curves of overall survival between high-risk group and low-risk group. **(B)** The prediction performance for three- and five-year overall survival by the ROC analysis.

To confirm the survival prediction power of the six-lncRNA signature, we tested the six-lncRNA signature in the validation cohort. Each patient of validation cohort was assigned a risk score by the six-lncRNA signature, and was classified into the high-risk or low-risk patient according to the threshold of the median risk score (0.562) derived from the training cohort. The patients of the validation cohort were classified as high-risk (n=59) or low-risk (n=53) with significantly different survival time. Kaplan-Meier curves for the high-risk and low-risk groups within the validation cohort are shown in Figure 2A. As in the training group, the overall survival time of the high-risk group patients was significantly shorter than that of low-risk group patients (median survival 72.8 months vs. 174.6 months, p=0.037) (Figure 2A). In details, the survival of patients in the low-risk group was 86.3% at 36 months and 77.5% at 60 months which compared with 69.9% and 55.2%, respectively, in the high-risk group. The univariable analysis also revealed a significant association between six-lncRNA signature risk score and overall survival (HR=1.434, 95% CI=1.091-1.885, p=0.01) (Table 2). The AUC of the six-lncRNA signature for survival prediction in the validation cohort was 0.621 at 36 months of OS and 0.623 at 60 months of OS. Distribution of the six-lncRNA signature risk scores, the expression pattern of prognostic lncRNAs and the survival status was shown in Supplementary Figure 1A, which is consistent with findings in the training cohort.

**Figure 3:**
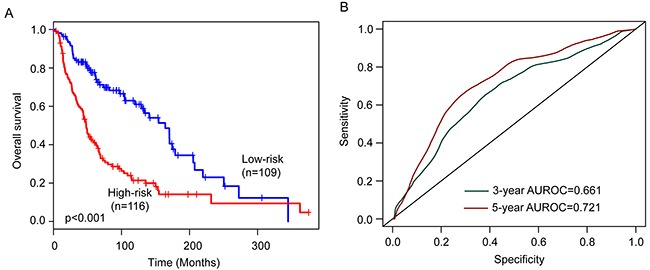
Performance validation of the six-lncRNA signature in the entire TCGA cohort **(A)** Kaplan-Meier survival curves of overall survival between high-risk group and low-risk group. **(B)** The prediction performance for three- and five-year overall survival by the ROC analysis.

The six-lncRNA signature was then tested for its prognostic value in the 225 patients of entire TCGA cohort. The same six-lncRNA signature and threshold as those derived from the training cohort classified 116 and 109 patients of the entire TCGA cohort into the high-risk and low-risk groups, respectively. Consistent with the findings described above, patients in the high-risk group had significantly shorter overall survival than those in the low-risk group (median survival 48 months vs. 164 months, p<0.001) (Figure 3A). In details, the survival of patients in the low-risk group was 84% at 36 months and 77.6% at 60 months which compared with 60.5% and 41%, respectively, in the high-risk group. The univariable analysis also revealed a significant association between six-lncRNA signature risk score and overall survival (Table 2). The AUC of the six-lncRNA signature for survival prediction was 0.661 at 36 months of OS and 0.721 at 60 months of OS.

Distribution of the six-lncRNA signature risk scores, the expression pattern of prognostic lncRNAs and the survival status was shown in Supplementary Figure 1B, which is consistent with findings in the training cohort and validation cohort.

### Independent prognostic value of the six-lncRNA signatures

To test whether the prognostic power of the six-lncRNA signature for survival prediction is independent of other clinicopathological factors, we performed multivariate Cox regression to test the performance of the six-lncRNA signature in comparison with other clinical factors, including age, gender and stage. The results from the training cohort showed that the six-lncRNA signature (HR=3.518, 95% CI=2.331-5.31, p<0.001), stage III (HR=2.214, 95% CI=1.098-4.467, p=0.026) and stage IV (HR=21.13, 95% CI=2.274-196.386, p=0.007) was significantly correlated with overall survival of the patients with cutaneous melanoma (Table 2). As shown in Table 2, in the validation cohort, multivariate analysis showed that the six-lncRNA signature (HR=1.62, 95% CI=1.178-2.231, p=0.003) and age (HR=1.02, 95% CI=1.004-1.042, p=0.018). Combined training and validation cohort showed that the six-lncRNA signature (HR=2.043, 95% CI=1.616-2.583, p<0.001), age (HR=1.017, 95% CI=1.003-1.03, p=0.014), stage III (HR=1.926, 95% CI=1.196-3.102, p=0.007) and stage IV (HR=3.21, 95% CI=1.298-7.941, p=0.012) was significant in the multivariate analysis.

**Figure 4:**
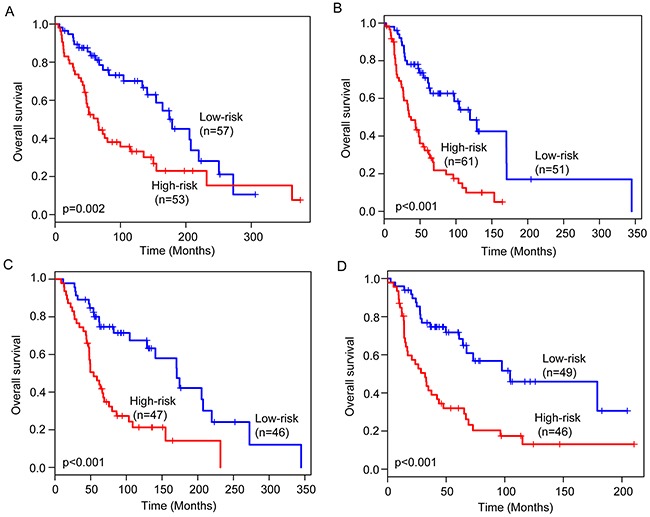
Stratification analysis for age and stage Kaplan-Meier survival curves of overall survival between high-risk group and low-risk group for younger patients **(A)** and elder patients **(B)**. Kaplan-Meier survival curves of overall survival between high-risk group and low-risk group for early-stage patients **(C)** and advanced-stage patients **(D).**

We next performed data stratification analysis for age and stage. With the six-lncRNA signature, the younger patients can be further subdivided into the high-risk group and low-risk group with significantly different survival (p=0.002) (Figure 4A). Similar results were observed when the six-lncRNA signature was applied to the elder patients (p<0.001) (Figure 4B). Then we stratified the entire TCGA patients into early-stage patients and advanced-stage patients. The six-lncRNA signature could classify early-stage patients into the high-risk group and low-risk group with significantly different survival (p<0.001) (Figure 4C). For advanced-stage patients, the six-lncRNA signature also showed similar prognostic value for survival prediction (Figure 4D). The results of the multivariate Cox regression and stratification analysis thus indicated that the predictive ability of the six-lncRNA signature is independent of other clinical factors for survival prediction in patients with cutaneous melanoma.

$$\text{lncRNA based risk score}=\sum i=1n(\exp_{i}*coef)$$

## DISCUSSION

Cutaneous melanoma is one of the most common malignancies and causes the greatest number of skin cancer-related deaths. Prediction of disease progression and prognosis is important for personalized risk assessment to improve treatment efficacy. With advances in the high-throughput omics study, large-scale genomic analyses of cutaneous melanoma have provided insights into the biological heterogeneity of cutaneous melanoma which has potentially important implications for improving prognosis [32]. Although traditional clinical and histological variables have been applied to guide treatment decisions, the early identification of patients at highest risk for disease progression still is unsatisfactory because of highly variable clinical behavior and molecular heterogeneity [4]. Increasing evidence in the molecular profiling analysis has found that molecular characterization can improve prognosis prediction compared with traditional clinical and histological variables. Timar reported a meta-analysis of the metastasis-gene signatures using seven melanoma cohorts from Gene Expression Omnibus (GEO) and identified a 350-gene signature [33]. Another study performed by Gerami *et al*. revealed a 28-gene signature to predict the metastatic risk associated with cutaneous melanoma [34]. Furthermore, recent some efforts also have been made to access the clinical significance of miRNAs in cutaneous melanoma and successfully developed several miRNA-based signatures to improve risk stratification for patients with cutaneous melanoma. For example, analysis of miRNA microarray expression profiling in primary and metastatic melanoma specimens identified a six-miRNA signature significantly stratified stage III patients into “better” and “worse” prognostic patients group [35]. A subsequent study of 80 melanoma patients at primary diagnosis identified a five-miRNA signature which can be used to determine recurrence risk of primary melanoma patients [36].

Despite great improvements in developing molecular biomarkers in cutaneous melanoma, these existing molecular signatures were mainly based on expression of mRNAs or miRNAs. Recently, a novel class of ncRNAs, termed lncRNAs, has been discovered and indicated as one of cancer hallmarks [37]. Compare to mRNAs and miRNAs, expression and functions of lncRNAs tended to be typically more specific in terms of cell-, tissue- and tumor-type [38]. Moreover, many lncRNAs were found to be stable and easily detectable in plasma or other body fluids, highlighting the possibilities of lncRNAs for the diagnostics and treatment of cancer [39]. Although several lncRNAs have been reported to be involved in the pathogenesis of melanoma, such as *BANCR, SLNCR1, CASC15, MALAT1* and so on [12], genome-wide systematic analysis for the predictive value of lncRNAs in prognosis prediction for cutaneous melanoma is lacking because of limited available lncRNA expression profiles in cutaneous melanoma. Fortunately, Li et al proposed a computational pipeline to obtained genome-wide lncRNA expression profiles in a large number of patients with various cancers by repurposing large-scale RNA-Seq cohorts from TCGA project [40], thus facilitating the discovery and validation of novel lncRNA biomarkers in some cancers and providing an unprecedented opportunity to systematically evaluate clinical implication for diagnosis and prognosis in cutaneous melanoma.

In this study, we tried to assess the prognostic value of lncRNAs in cutaneous melanoma by integrated lncRNA expression profiles from TCGA database and matched clinical information from a large cohort of patients with cutaneous melanoma. We finally identified a set of six lncRNAs that are significantly associated with the survival of patients with cutaneous melanoma. A linear combination of six lncRNAs (*LINC01260, HCP5, PIGBOS1, RP11-247L20.4, CTA-292E10.6* and *CTB-113P19.5*) was constructed as an indicator for the clinical outcome of patients with cutaneous melanoma. By applying the six-lncRNA signature to the training cohort, a clear separation was observed in the survival curves between high-risk group and the low-risk group. Patients with high-risk lncRNA signature had poor survival outcome than those with low-risk lncRNA signature. Moreover, the six-lncRNA signature demonstrated robust and stable prognostic ability for survival prediction in both the validation cohort and entire TCGA cohort. Further analysis of univariable and multivariate Cox regression models showed that the six- lncRNA signature was an independent prognostic factor for patients with cutaneous melanoma. The six lncRNAs identified in this study provide novel insights into the molecular heterogeneity of cutaneous melanoma and also have potentially important implications for prognosis and therapy for cutaneous melanoma.

Although our results highlight the potential of lncRNA expression profiling to improve clinical prognosis in patients with cutaneous melanoma, some limitations should be recognized. Firstly, the six lncRNAs was discovered and validated in the patients from a single source (TCGA project) and their prognostic values should be tested in another independent patient cohort. Secondly, the biological function of these six lncRNAs has not been reported up until now and therefore needed to be studied using biological experiments in the future.

## MATERIALS AND METHODS

### Clinical characteristics of patients with cutaneous melanoma

Clinical characteristics of patients with cutaneous melanoma were downloaded from The Cancer Genome Atlas (TCGA) data portal (https://cancergenome.nih.gov/). After removing patients without clinical information and lncRNA expression profiles, 225 patients with cutaneous melanoma were used in this study. Patients with cutaneous melanoma were randomly divided into a training cohort (n=113) and a validation cohort (n=112). The detailed clinical characteristics of patients in the training cohort and validation cohort were summarized in Table 3.

**Table 3:** Clinical characteristics of patients with cutaneous melanoma in the three cohorts

Variables		Training cohort	Testing cohort	Combined cohort
		(n=113)	(n=112)	(n=225)
Vital status, n(%)	Alive	43(38.1)	43(38.4)	86(38.2)
	Dead	70(61.9)	69(61.6)	139(61.8)
Age years, n(%)	>=60	41(36.3)	53(47.3)	94(41.8)
	<60	72(63.7)	56(50.0)	128(56.9)
	NA	0	3(2.7)	3(1.3)
Gender, n(%)	Female	42(37.2)	42(37.5)	84(37.3)
	Male	71(62.8)	70(62.5)	141(62.7)
Stage, n(%)	Stage I/II	46(40.7)	47(42.0)	93(41.3)
	Stage III/IV	50(44.3)	45(40.1)	95(42.2)
	NA	17(15.0)	20(17.9)	37(16.5)

### Genome-wide lncRNA expression profiles of patients with cutaneous melanoma

Genome-wide lncRNA expression profiles of patients with cutaneous melanoma were obtained from the TANRIC database (http://bioinformatics.mdanderson.org/) [40]. Briefly, the genomic coordinates of 13,870 human lncRNAs from the GENCODE Resource (version 19) were obtained and were further filtered by removing those lncRNAs whose exons overlapped with any known coding genes based on the gene annotations of GENCODE. Expression levels of the remaining lncRNAs were measured as reads per kilobase per million mapped reads (RPKM) [40]. Then we removed lncRNA with RPKM expression values of 0 in >10% tumor samples. Finally, 3100 lncRNAs were retained for further study.

### Definition of prognostic lncRNA signature

Univariable Cox regression analysis was used to identify candidate lncRNAs that are significantly associated with survival. Then these candidate lncRNAs were subjected to the multivariate Cox regression analysis to access their interactive effect and identify independent prognostic lncRNAs to predict survival of patients. To develop a lncRNA signature, these independent prognostic lncRNAs were fitted in a multivariate Cox regression model in the training cohort to obtain estimated regression coefficients as weights to represent their relative power in predicting survival. Then a prognostic lncRNA signature was constructed by including expression values of each prognostic lncRNAs, weighted by their estimated regression coefficients in the multivariate Cox regression analysis as follows:

Where n is the number of lncRNAs in this signature, exp_*i*_ is the expression value of lncRNA_i_ and *Coef* is the estimated regression coefficients of lncRNA_i_ in the multivariate Cox regression analysis. The median risk score in the training cohort was used to as risk cutoff value to classify patients into the high-risk group and low-risk group.

### Survival analysis

Kaplan-Meier survival curves and log-rank tests were used to assess the differences in survival time between the high-risk and low-risk patients using the R package “survival”. Multivariate analyses were performed using Cox proportional hazards regression model to determine whether the lncRNA signature was independent of other clinical variables. Hazard ratios (HR) and 95% confidence intervals (CI) were calculated. The prognostic accuracy of lncRNA signature was also tested using time-dependent receiver operating characteristic (ROC) analysis using the R package“survival-ROC”[41]. We used the area under the curve at three and five years to measure prognostic accuracy. All analyses were performed using the R/Bio-Conductor (version 3.0.2).

## SUPPLEMENTARY MATERIALS FIGURES



## References

[1] Wortsman X (2012). Sonography of the primary cutaneous melanoma: a review. Radiol Res Pract.

[2] Siegel RL, Miller KD, Jemal A (2016). Cancer statistics, 2016. CA Cancer J Clin.

[3] Hanniford D, Zhong J, Koetz L, Gaziel-Sovran A, Lackaye DJ, Shang S, Pavlick A, Shapiro R, Berman R, Darvishian F, Shao Y, Osman I, Hernando E (2015). A miRNA-based signature detected in primary melanoma tissue predicts development of brain metastasis. Clin Cancer Res.

[4] Dickson PV, Gershenwald JE (2011). Staging and prognosis of cutaneous melanoma. Surg Oncol Clin N Am.

[5] Rajkumar S, Watson IR (2016). Molecular characterisation of cutaneous melanoma: creating a framework for targeted and immune therapies. Br J Cancer.

[6] Consortium ENCODE Project, Birney E, Stamatoyannopoulos JA, Dutta A, Guigo R, Gingeras TR, Margulies EH, Weng Z, Snyder M, Dermitzakis ET, Thurman RE, Kuehn MS, Taylor CM (2007). Identification and analysis of functional elements in 1% of the human genome by the ENCODE pilot project. Nature.

[7] Gibb EA, Brown CJ, Lam WL (2011). The functional role of long non-coding RNA in human carcinomas. Mol Cancer.

[8] Mercer TR, Mattick JS (2013). Structure and function of long noncoding RNAs in epigenetic regulation. Nat Struct Mol Biol.

[9] Gibb EA, Vucic EA, Enfield KS, Stewart GL, Lonergan KM, Kennett JY, Becker-Santos DD, MacAulay CE, Lam S, Brown CJ, Lam WL (2011). Human cancer long non-coding RNA transcriptomes. PLoS One.

[10] Sun J, Shi H, Wang Z, Zhang C, Liu L, Wang L, He W, Hao D, Liu S, Zhou M (2014). Inferring novel lncRNA-disease associations based on a random walk model of a lncRNA functional similarity network. Mol Biosyst.

[11] Zhou M, Wang X, Li J, Hao D, Wang Z, Shi H, Han L, Zhou H, Sun J (2015). Prioritizing candidate disease-related long non-coding RNAs by walking on the heterogeneous lncRNA and disease network. Mol Biosyst.

[12] Hulstaert E, Brochez L, Volders PJ, Vandesompele J, Mestdagh P (2017). Long non-coding RNAs in cutaneous melanoma: clinical perspectives. Oncotarget.

[13] Leucci E, Coe EA, Marine JC, Vance KW (2016). The emerging role of long non-coding RNAs in cutaneous melanoma. Pigment Cell Melanoma Res.

[14] Cao W, Liu JN, Liu Z, Wang X, Han ZG, Ji T, Chen WT, Zou X (2017). A three-lncRNA signature derived from the Atlas of ncRNA in cancer (TANRIC) database predicts the survival of patients with head and neck squamous cell carcinoma. Oral Oncol.

[15] Fan ZY, Liu W, Yan C, Zhu ZL, Xu W, Li JF, Su L, Li C, Zhu ZG, Liu B, Yan M (2016). Identification of a five-lncRNA signature for the diagnosis and prognosis of gastric cancer. Tumour Biol.

[16] Li J, Chen Z, Tian L, Zhou C, He MY, Gao Y, Wang S, Zhou F, Shi S, Feng X, Sun N, Liu Z, Skogerboe G (2014). LncRNA profile study reveals a three-lncRNA signature associated with the survival of patients with oesophageal squamous cell carcinoma. Gut.

[17] Song P, Jiang B, Liu Z, Ding J, Liu S, Guan W (2017). A three-lncRNA expression signature associated with the prognosis of gastric cancer patients. Cancer Med.

[18] Wang W, Yang F, Zhang L, Chen J, Zhao Z, Wang H, Wu F, Liang T, Yan X, Li J, Lan Q, Wang J, Zhao J (2016). LncRNA profile study reveals four-lncRNA signature associated with the prognosis of patients with anaplastic gliomas. Oncotarget.

[19] Yang K, Hou Y, Li A, Li Z, Wang W, Xie H, Rong Z, Lou G, Li K (2017). Identification of a six-lncRNA signature associated with recurrence of ovarian cancer. Sci Rep.

[20] Zeng JH, Liang L, He RQ, Tang RX, Cai XY, Chen JQ, Luo DZ, Chen G (2017). Comprehensive investigation of a novel differentially expressed lncRNA expression profile signature to assess the survival of patients with colorectal adenocarcinoma. Oncotarget.

[21] Zhou M, Sun Y, Sun Y, Xu W, Zhang Z, Zhao H, Zhong Z, Sun J (2016). Comprehensive analysis of lncRNA expression profiles reveals a novel lncRNA signature to discriminate nonequivalent outcomes in patients with ovarian cancer. Oncotarget.

[22] Sun J, Chen X, Wang Z, Guo M, Shi H, Wang X, Cheng L, Zhou M (2015). A potential prognostic long non-coding RNA signature to predict metastasis-free survival of breast cancer patients. Sci Rep.

[23] Sun J, Cheng L, Shi H, Zhang Z, Zhao H, Wang Z, Zhou M (2016). A potential panel of six-long non-coding RNA signature to improve survival prediction of diffuse large-B-cell lymphoma. Sci Rep.

[24] Zhou M, Diao Z, Yue X, Chen Y, Zhao H, Cheng L, Sun J (2016). Construction and analysis of dysregulated lncRNA-associated ceRNA network identified novel lncRNA biomarkers for early diagnosis of human pancreatic cancer. Oncotarget.

[25] Zhou M, Guo M, He D, Wang X, Cui Y, Yang H, Hao D, Sun J (2015). A potential signature of eight long non-coding RNAs predicts survival in patients with non-small cell lung cancer. J Transl Med.

[26] Zhou M, Wang X, Shi H, Cheng L, Wang Z, Zhao H, Yang L, Sun J (2016). Characterization of long non-coding RNA-associated ceRNA network to reveal potential prognostic lncRNA biomarkers in human ovarian cancer. Oncotarget.

[27] Zhou M, Xu W, Yue X, Zhao H, Wang Z, Shi H, Cheng L, Sun J (2016). Relapse-related long non-coding RNA signature to improve prognosis prediction of lung adenocarcinoma. Oncotarget.

[28] Zhou M, Zhao H, Wang Z, Cheng L, Yang L, Shi H, Yang H, Sun J (2015). Identification and validation of potential prognostic lncRNA biomarkers for predicting survival in patients with multiple myeloma. J Exp Clin Cancer Res.

[29] Zhou M, Zhao H, Xu W, Bao S, Cheng L, Sun J (2017). Discovery and validation of immune-associated long non-coding RNA biomarkers associated with clinically molecular subtype and prognosis in diffuse large B cell lymphoma. Mol Cancer.

[30] Zhou M, Zhong L, Xu W, Sun Y, Zhang Z, Zhao H, Yang L, Sun J (2016). Discovery of potential prognostic long non-coding RNA biomarkers for predicting the risk of tumor recurrence of breast cancer patients. Sci Rep.

[31] Zhou M, Zhang Z, Zhao H, Bao S, Cheng L, Sun J (2017). An immune-related six-lncRNA signature to improve prognosis prediction of glioblastoma multiforme. Mol Neurobiol.

[32] Cancer Genome Atlas Network (2015). Genomic classification of cutaneous melanoma. Cell.

[33] Timar J, Gyorffy B, Raso E (2010). Gene signature of the metastatic potential of cutaneous melanoma: too much for too little?. Clin Exp Metastasis.

[34] Gerami P, Cook RW, Wilkinson J, Russell MC, Dhillon N, Amaria RN, Gonzalez R, Lyle S, Johnson CE, Oelschlager KM, Jackson GL, Greisinger AJ, Maetzold D (2015). Development of a prognostic genetic signature to predict the metastatic risk associated with cutaneous melanoma. Clin Cancer Res.

[35] Segura MF, Belitskaya-Levy I, Rose AE, Zakrzewski J, Gaziel A, Hanniford D, Darvishian F, Berman RS, Shapiro RL, Pavlick AC, Osman I, Hernando E (2010). Melanoma microRNA signature predicts post-recurrence survival. Clin Cancer Res.

[36] Friedman EB, Shang S, de Miera EV, Fog JU, Teilum MW, Ma MW, Berman RS, Shapiro RL, Pavlick AC, Hernando E (2012). Serum microRNAs as biomarkers for recurrence in melanoma. J Transl Med.

[37] Gutschner T, Diederichs S (2012). The hallmarks of cancer: a long non-coding RNA point of view. RNA Biol.

[38] Cheetham SW, Gruhl F, Mattick JS, Dinger ME (2013). Long noncoding RNAs and the genetics of cancer. Br J Cancer.

[39] Huarte M (2015). The emerging role of lncRNAs in cancer. Nat Med.

[40] Li J, Han L, Roebuck P, Diao L, Liu L, Yuan Y, Weinstein JN, Liang H (2015). TANRIC: an interactive open platform to explore the function of lncRNAs in cancer. Cancer Res.

[41] Heagerty PJ, Lumley T, Pepe MS (2000). Time-dependent ROC curves for censored survival data and a diagnostic marker. Biometrics.

