# Supplementary material for: Expression profiles analysis of long non-coding RNAs identified novel lncRNA biomarkers with predictive value in outcome of cutaneous melanoma
Source: Oncotarget. 2017 Sep 8;8(44):77761–70. doi: 10.18632/oncotarget.20780 (PMC5652813; doi:10.18632/oncotarget.20780)
Supplement: Supplementary file 1 [file oncotarget-08-77761-s001.pdf]

Expression profiles analysis of long non-coding RNAs identified novel lncRNA biomarkers with predictive value in outcome of cutaneous melanoma

SUPPLEMENTARY MATERIALS

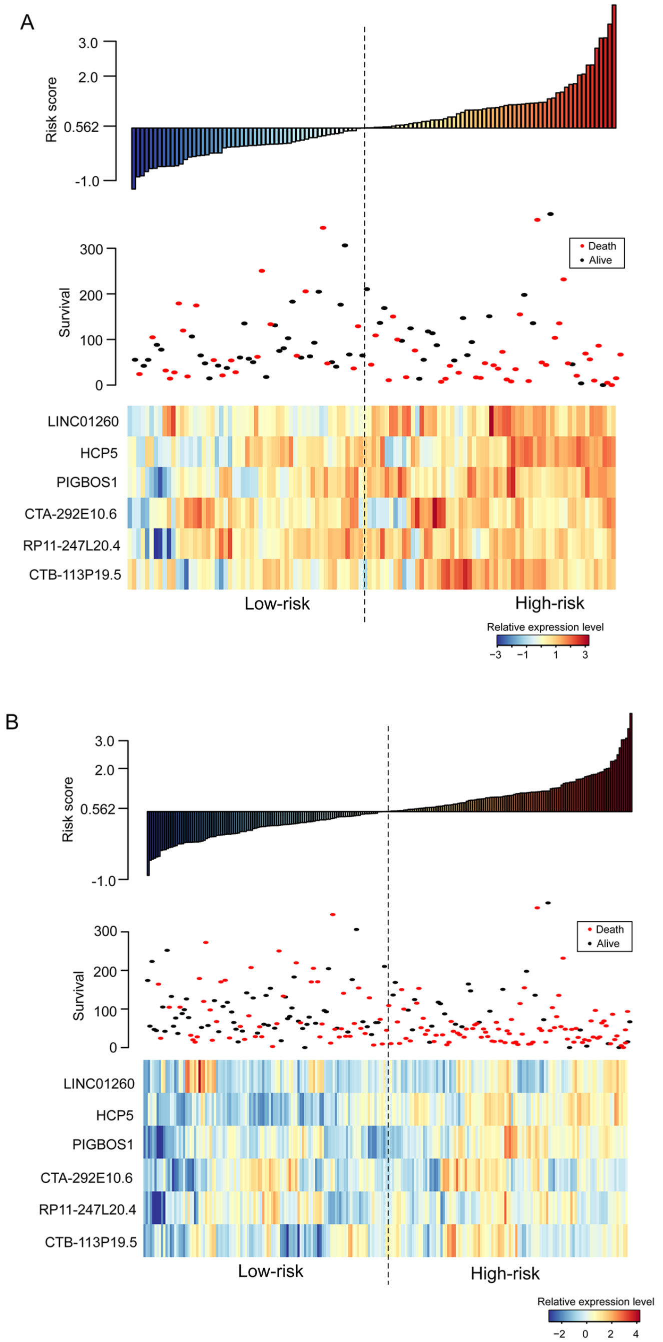

Supplementary Figure 1: Distribution of risk scores, patient survival status and lncRNA expression map in the validation cohort **(A)** and the entire TCGA cohort **(B)**.
